# Household chaos: a risk factor for adverse child outcomes gains attention in public health

**DOI:** 10.1186/s12889-020-08680-y

**Published:** 2020-05-01

**Authors:** Jennifer A. Emond

**Affiliations:** 1 The Department of Biomedical Data Science and the Department of Pediatrics, Geisel School of Medicine, Dartmouth College, Hanover, NH USA; 2 One Medical Center Drive, Hinman Box 7920, Rubin 829, Hanover, NH 03755 USA

## Abstract

Household chaos, characterized by high levels of confusion, disorganization and hurriedness in the home, is increasingly recognized as an important risk factor for adverse child outcomes. Early research on household chaos and child well-being was largely within the field of developmental psychology, where greater levels of household chaos has been associated with greater behavioral, attention and learning problems in young children. The potential influence of household chaos on child health behaviors is more recently gaining attention within public health. A recent study by Marsh et al., entitled, the *Relationship between Household Chaos and Child, Parent, and Family Outcomes: A Systematic Scoping Review,* presents the findings from 112 studies that assessed the influence of household chaos on a wide range of child outcomes. Findings highlight the various adverse child health outcomes across multiple domains that may be negatively affected by greater levels of household chaos including a few that reflect child health behaviors such as sleep, diet and weight gain. The review additionally presents findings from mediation and moderation analyses. This commentary highlights key aspects of the Marsh et al. review and outlines the implications of the work within health behavior research. This commentary further identifies child screen media use as a critically understudied area when considering the interplay between household chaos and child well-being.

There is a growing awareness that household chaos may negatively impact child development and well-being. Household chaos is characterized by high levels of confusion, disorganization and hurriedness in the home. While short-term bouts of chaos in the home are normal, chronic exposure to household chaos can have a profound impact on child development. Early research on household chaos and child well-being has largely been within the field of developmental psychology, and greater levels of household chaos has been associated with greater behavioral, attention and learning problems [1–4] in young children. Attention is more recently being given to the importance of household chaos on child health outcomes traditionally associated with health behavior research and public health. For example, greater household chaos has been independently related to poor asthma control among children and adolescents [5] and poor glycemic control among adolescents with type 1 diabetes [6]. Household chaos has also been identified as a potential obesity risk factor during infancy [7] and childhood [8]. Importantly, the associations between household chaos and child well-being across the available studies account for confounding measures such as sociodemographic characteristics, suggesting that household chaos is a unique construct that relates to child well-being.

In an article published in *BMC Public Health*, Marsh and colleagues [9] promote the awareness of household chaos as a potential influence on child well-being across domains by presenting a comprehensive, scoping review of studies examining household chaos and child well-being. The authors included 112 analyses from 111 published studies; 60 (53.6%) studies were cross-sectional, 49 (43.8%) were longitudinal, 2 (1.8%) included an experimental design and 1 (0.9%) was a case-control study. Household chaos was largely operationalized using validated scales, such as the Confusion, Hubbub and Order Scale developed by Matheny et al. [10] The Matheny scale is a 15-item scale that has been validated against objective assessments within homes with young children. Child outcomes captured in the Marsh et al. review included cognitive and academic performance, socio-emotional and behavioral problems (e.g., externalizing behaviors), communication and language development, parenting style and family characteristics, as well as outcomes related to child health behaviors including child weight status, eating behaviors, glycemic control and sleep. Eight studies also assessed biomarkers including child cortisol concentrations and gut biomarkers.

Overall, findings demonstrated that household chaos was associated with multiple adverse child outcomes including those aligned with health behavior research such as increased weight status, poor sleep and poor dietary behaviors. For example, greater levels of household chaos was associated with greater self-reported barriers to preparing meals at home among parents, less availability of healthy food at home, and fewer meals together as a family in a sample of 819 households with young children [11]. Household chaos was also reported to act as a moderator in a few studies, where the impact of risk factors on adverse child outcomes was more pronounced among the most chaotic homes. For example, in a twin study of 3258 monozygotic and dizygotic twin pairs and their parents [12], the presence of depressive symptoms at age 12 years was heritable and that genetic risk was greatest for children in the most chaotic homes.

A particularly interesting aspect of the Marsh et al. review is that findings suggest household chaos may act as a mediator of the relationship between risk factors and adverse child outcomes. Sixteen studies included in the review assessed household chaos as a mediator and findings largely supported partial mediation. For example, the detrimental effect of low household income at age 2–3 years on emotional problems at age 7–8 years was partially mediated by higher household chaos at age 4–5 years in a study among children as they aged from 2 to 8 years [13]. Conversely, authors also summarized studies that assessed if the main effect of household chaos on adverse child outcomes was mediated by other risk factors. Eleven studies assessed whether other factors mediated the association between household chaos and adverse child outcomes and several studies reported null associations. For example, infant breastfeeding, screen use or sleep did not mediate the association between greater household chaos, averaged at age 6 and 12 months, and greater weight-for-length z score at age 12 months among a sample of 401 infants as they aged from 6 to 12 months [7]. It is informative for studies to report null findings for mediation analyses given that the direction of causality between household chaos and other child, parent and household risk factors affecting child well-being remain unknown. Yet it is unclear whether publication bias may have affected the studies included in the review. However, results demonstrate the different conceptual models across the available studies examining household chaos and child well-being, and highlight the need for longitudinal study designs with household chaos assessed at multiple time points to assess causality.

The implications of the research on household chaos span across multiple disciplines including public health. Clinicians and child health advocates should consider the influence of household chaos on child well-being when interacting with families in a clinical setting or when advocating for programs and policies to reduce the burden highly chaotic homes may uniquely face. Health behavior researchers additionally should consider the role of household chaos when examining risk factors for suboptimal child health behaviors, including poor diet, obesity risk, screen use, short sleep and substance use. Importantly, the design of health behavior interventions should consider the capacity of highly chaotic homes to adhere to intervention activities. For example, how can incentive programs be designed to best support chaotic homes marked by a lack of structure and routine?

The availability of validated scales of household chaos enables researchers to identify families that likely need additional support in adhering to health behavior interventions. It is important to note that the available chaos scales are not diagnostic, and currently no threshold exists to define a chaotic versus a non-chaotic home. However, routinely including household chaos measures in behavioral intervention studies and using standardized scoring metrics can help identify families at risk of non-adherence. It will also be important to identify if household chaos is a mediator between socio-demographic characteristics, such as lower household income, and parent or child adherence to health behavior interventions. That information will help avoid generalizations about socio-demographic risk factors and child health, and findings can inform the design of support systems to appropriately help families in their efforts at health behavior change.

The review by Marsh et al. also highlights areas within public health that should further investigate the role of household chaos on child well-being. For example, research on household chaos and digital media use among children is scant [8, 14]. The Matheny measure of household chaos is independent of child and household media characteristics (in comparison to other measures of household chaos that did include television use in the home [2]), and household chaos was strongly and linearly associated with parent-reported child media use among a sample of 385 parents with children age 2–5 years using that Matheny scale [14]. Greater levels of media use is associated with multiple adverse outcomes among children including poor self-regulation [15], poor sleep, inactivity and poor diet [16], and media use can expose children to inappropriate content including substance use [17, 18]. It is also possible that excessive social media use in adolescence may relate to internalizing behaviors including depression and anxiety [19, 20] and risky sexual behaviors [18, 21, 22], although findings are mixed [23]. Thus, it will be critically important for future research to examine the inter-dependent and potentially causal associations between household chaos, child media use and adverse child health outcomes, and to identify sustainable interventions to help more chaotic homes adhere to media use limits for their children.

In summary, household chaos appears to be a unique construct that is associated with multiple areas of children development, health and well-being. Health behavior researchers should consider the role of household chaos on adverse child outcomes and consider the influence of household chaos on a family’s ability to adhere to health behavior interventions. Future work is needed to standardize findings across available studies to more comprehensively understand the influence of household chaos on child well-being across multiple domains. Regardless, current findings highlight the need to consider household chaos when assessing child well-being and provide the impetuses for future studies to assess causal associations.

## Data Availability

Not applicable.
